# Cefepime neurotoxicity in elderly patient with renal impairment: a case report

**DOI:** 10.1097/MS9.0000000000004715

**Published:** 2026-01-21

**Authors:** Muhammad Furqan, Abdullah Saeed, Sidrah Khan, Muhammad Usman Haider, Sadia Binte Rahim

**Affiliations:** a Department of Medicine, King Edward Medical University, Lahore, Pakistan; b Department of Internal Medicine, Geisinger Health System, Wilkes-Barre, Pennsylvania; c Department of Medicine, Mymensingh Medical College Hospital, Mymensingh, Bangladesh

**Keywords:** altered mental status, broad-spectrum antibiotics, cefepime neurotoxicity, FDA drug safety, renal impairment, sepsis, subarachnoid hemorrhage, ventriculoperitoneal shunt

## Abstract

**Introduction and importance:**

While cefepime-induced neurotoxicity is a known complication of beta-lactam therapy in renal impairment, its diagnosis is uniquely challenging in patients with complex neurosurgical baselines. This case illustrates the risk of diagnostic anchoring where the presence of a ventriculoperitoneal shunt diverts clinical suspicion toward mechanical failure and obscures a reversible toxic etiology.

**Case presentation:**

A 63-year-old female with subarachnoid hemorrhage, slit ventricle syndrome, and a ventriculoperitoneal shunt presented with septic shock and acute kidney injury. Following renal adjustment of cefepime for *Pseudomonas* bacteremia, she developed profound altered mental status. Initial evaluation prioritized shunt malfunction, yet computed tomography was confounded by chronic slit ventricle physiology. Electroencephalography revealed generalized triphasic waves characteristic of metabolic encephalopathy rather than structural dysfunction.

**Clinical discussion:**

Cefepime-induced neurotoxicity (CIN) pathophysiology involves gamma-aminobutyric acid-A receptor inhibition amplified by reduced renal clearance and sepsis-induced blood–brain barrier disruption. Here, the clinical picture was masked by the overlap between toxic encephalopathy and insidious shunt failure symptoms. This necessitated distinguishing toxicity from nonconvulsive status epilepticus or hydrocephalus using electroencephalogram (EEG), defying the heuristic that neurological decline in shunted patients is mechanical until proven otherwise.

**Conclusion:**

This case underscores that CIN can mimic mechanical shunt failure, which necessitates a high index of suspicion in patients with indwelling neurosurgical hardware. Early EEG utilization is critical to overcome diagnostic bias and ensure prompt antibiotic discontinuation rather than unnecessary neurosurgical intervention.

## Introduction

Cefepime, a fourth-generation cephalosporin, is used for its ability to target a broad spectrum of organisms, including *Pseudomonas aeruginosa*. It exerts its bactericidal action by inhibiting bacterial cell wall synthesis. Being resistant to degradation by beta-lactamases, cefepime can be used against a wide variety of microbes, thereby limiting the need for carbapenems^[1,2]^. While cefepime-induced neurotoxicity (CIN) is generally uncommon, a systematic review estimated a minimum incidence of approximately 1 per 480 treatment courses, with 87% of affected patients demonstrating underlying renal dysfunction^[3]^. Additionally, a therapeutic drug-monitoring cohort study with measured trough levels reported neurotoxicity in 23% of cefepime-exposed patients, with reduced estimated glomerular filtration rate emerging as the strongest independent predictor of toxicity^[4]^. Clinically, this neurotoxicity universally presents with altered mental status, most commonly manifesting as reduced consciousness and confusion alongside myoclonus, with symptoms typically emerging after a latency period of 2–5 days following drug initiation^[5]^. Prompt discontinuation of the drug leads to cessation of symptoms; however, prevention and early recognition of warning signs are the best approaches to avoid unnecessary investigations and treatments. Here, we present a case of an older woman with a complex medical history who developed neurotoxic effects due to cefepime use in the critical care setting.

**HIGHLIGHTS**

- Cefepime toxicity can mimic shunt blockage, confusing the diagnosis in patients with brain shunts.
- Sepsis-induced blood–brain barrier disruption synergizes with renal impairment to accelerate central nervous system drug accumulation.
- Electroencephalogram definitively differentiates metabolic encephalopathy from structural progression when neuroimaging is confounded by slit ventricle syndrome.
- Generalized triphasic waves distinguish toxic antibiotic effects from nonconvulsive status epilepticus to guide appropriate deprescribing.
- Early recognition and drug cessation prevent unnecessary neurosurgical interventions in patients with complex intracranial baselines.

## Methods

Our case report adheres to the CARE guidelines^[6]^. To ensure transparency about our case report, we note that this work complies with the TITAN (Transparency In The reporting of Artificial INtelligence) 2025 guidelines^[7]^. All use of AI (Artificial Intelligence) tools in drafting and revising the text has been explicitly declared and reviewed in accordance with those recommendations.

## Case presentation

Our patient, a 63-year-old female with previous medical history significant for hypertension, type 2 diabetes mellitus, hyperlipidemia, hypothyroidism, chronic right foot drop and subarachnoid hemorrhage (SAH) due to ruptured AV fistula (requiring ventriculoperitoneal shunt [VPS] placement), presented to the emergency department due to sudden inability to void since the previous night along with an episode of vomiting. There were no associated headaches, motor weakness, or visual changes. Laboratory and clinical findings on admission are given in Table 1.

**Table 1:** Laboratory and clinical findings on admission

Parameter	Patient value	Reference range	Clinical interpretation
Systolic blood pressure	60–70 mmHg	90–120 mmHg	Profound hypotension
Heart rate	Up to 182 bpm	60–100 bpm	Marked tachycardia
WBC count	59.48 × 10^9^/L	4.0–10.0 × 10⁹/L	Severe leukocytosis
Lactate	10.2 mmol/L	0.5–2.0 mmol/L	Tissue hypoperfusion
C-reactive protein	120 mg/L	<5.0 mg/L	Significant inflammation
Serum creatinine	2.2 mg/dL	0.6–1.3 mg/dL	Acute kidney injury
Estimated GFR	24 mL/min/1.73 m^2^	>60 mL/min/1.73 m^2^	Reduced renal function

Urology was consulted, and the patient underwent cystourethroscopy with bilateral retrograde pyelogram and bilateral ureteral stent insertion on 22 February 2025, for obstructing stones on the right and possible left ureteral stone/hydronephrosis. She was admitted to the ICU (Intensive Care Unit) with septic shock, with Proteus UTI (Urinary Tract Infection) and Pseudomonas bacteremia. She also developed diarrhea and was found to be *Clostridioides difficile* positive and was started on P.O. Vancomycin. Blood cultures drawn on the day of admission were positive for Pseudomonas and Proteus, and the patient was sensitive to Unasyn (Ampicillin–Sulbactam) and Cefepime. Therefore, the patient was started on cefepime.

Patient’s past medical history was significant for SAH due to a ruptured AV fistula back in 2016, for which she had occipital craniotomy and VPS placement. The shunt was replaced in February 2017 with a Medtronic Strata (most recent shunt setting: 1.5). Notably, the patient was previously admitted in August 2024 for urosepsis, in addition to a *C. difficile* infection. During workup for her altered mental status, she was incidentally noted to have chronic slit ventricles and small subdural fluid collections. The VP shunt was increased from 0.5 to 1.0 and then to 1.5, with some improvement in ventricular size (stable subdural collections), and the patient was discharged. She was again seen in the ED in October 2024 for weakness, confusion, and eye deviation. The computed tomography (CT) scan head showed a significant increase in ventricular size compared to the last CT head, with a decrease in the size of the bilateral subdural collections. The shunt was changed from 1.5 to 1.0. The patient was followed as an outpatient and ultimately underwent shunt revision on 23 December 2024 (Codman Certas set at 1.0). According to her husband at the bedside, the patient had done well following that surgery.

For altered mental status during the patient’s current admission, neurosurgery was consulted, given her recent shunt surgery and current presentation suggestive of septic shock. A CT scan of the head showed an incidental finding of cerebral ventricular over-shunting. On repeat CT angiography of the head, the patient had decreasing subdural collections with an increase in ventricular size (Codman Certas at 2). Repeat blood cultures on 24 February 2025, showed no growth; however, the Department of Infectious Diseases recommended Cefepime and Omnicef (Cefdinir) for three more days upon discharge. The Foley catheter was removed on 27 February, and the patient was downgraded to medicine/surgery from the PCU Progressive Care Unit the next day.

On 28 February, the patient presented with new-onset altered mental status. An infectious workup was negative, and a repeat CT head remained stable. Given the unexplained neurological decline, an electroencephalogram (EEG) was obtained. It revealed an abnormal background with generalized slowing composed predominantly of mixed theta frequencies, indicating diffuse cerebral dysfunction. Continuous attenuation was observed over the left temporal region, and a breach rhythm was noted in the right parietal area consistent with the known skull defect. Notably, frequent generalized triphasic waves were present, a pattern highly specific to metabolic or toxic encephalopathies. No seizures were captured. These findings strongly supported a diagnosis of toxic-metabolic encephalopathy, most compatible with CIN.

**Figure 1.**
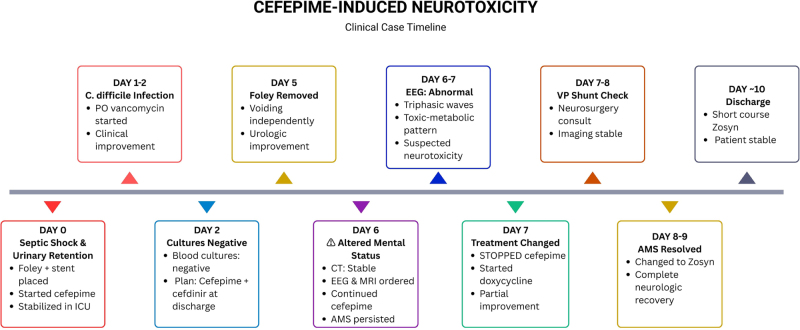
Chronological summary of clinical events highlighting the onset, diagnosis, and resolution of cefepime-induced neurotoxicity.

Cefepime was discontinued on 1 March, and the patient was switched to Doxycycline. An MRI and further monitoring were ordered to rule out alternative diagnoses (see Table 2 for differential diagnoses). The patient’s mental status subsequently improved, and the antibiotic regimen was optimized to Piperacillin-Tazobactam (Zosyn). The patient was discharged with scheduled neurosurgery follow-up. The complete clinical timeline of the hospital course is illustrated in Figure 1.

**Table 2:** Differential diagnosis

Differential	Rationale	Description
Cefepime-induced neurotoxicity	The onset of AMS after cefepime is typical in renal impairment	AMS resolved after cefepime was discontinued
Hospital-acquired delirium	ICU stay, infection, and advanced age	No other delirium triggers identified; resolved post-cefepime
Subclinical seizures	AMS without convulsions	Long-term EEG monitoring ordered
Stroke	Sudden-onset AMS	The MRI of the brain was unremarkable
Shunt malfunction or over-drainage	Recent shunt adjustments; prior history	CT head stable; neurosurgery signed off

## Discussion

Cephalosporins are generally considered safe for use in both gram-positive and gram-negative bacterial infections, owing to their wide therapeutic window, along with other drugs of this class^[8]^. However, along with other beta-lactam antibiotics, they have recently gained attention due to their propensity to cause neurotoxicity, particularly in critically ill patients.

The beta-lactam ring in cefepime is structurally similar to the gamma-aminobutyric acid (GABA) neurotransmitter in the CNS. Under normal physiological conditions, GABA binding to the GABA-A receptor complex induces the opening of chloride ion channels, resulting in neuronal membrane hyperpolarization and suppression of action potential generation. The underlying mechanism of CIN is believed to be due to its ability to cross the blood–brain barrier (BBB) and concentration-dependent competitive inhibition of the GABA_A_ receptor complex in the central nervous system (CNS), where it acts as a competitive antagonist that blocks endogenous GABA binding and prevents inhibitory chloride influx, effectively removing a critical inhibitory restraint on neuronal firing. Beta-lactam antibiotics have also been shown to decrease endogenous GABA release, along with an increase in the release of excitatory neurotransmitters^[9]^, and emerging evidence suggests a multi-hit mechanism whereby cefepime may additionally augment the release of excitatory amino acids such as glutamate, creating a synergistic state of excitotoxicity. The net effect of this is CNS hyperexcitation, a pathophysiological state distinct from the global neuronal depression observed in uremic or septic encephalopathy, which explains the predominance of “positive” neurological phenomena in CIN. Clinically, this hyperexcitability manifests along a dose-dependent spectrum ranging from confusion, obtundation, movement disorders, encephalopathy, myoclonus, seizures, to psychiatric features such as anxiety, panic attacks, hallucinations, and psychosis^[10,11]^. To mitigate such risks in future pharmacotherapy, the integration of advanced computational models offers a promising direction for early detection of toxicity. Recent informatics analyses highlight the revolutionary impact of AI models, particularly AlphaFold, in structural biology and drug discovery. By leveraging models like AlphaFold, researchers could potentially model the binding patterns between antibiotics and neuroreceptors (such as the GABAA complex) during the drug design phase. This predictive approach would allow for the assessment of neurotoxicity risks in advance, driving the development of safer antibiotics and reducing the incidence of adverse events in vulnerable populations^[12]^.

In terms of pharmacokinetics, cefepime undergoes renal clearance; hence, patients with impaired renal function are prone to developing higher serum concentrations of this drug and other organic acids and urea, which results in increased permeability across the BBB, leading to neurotoxic effects^[4]^. In the setting of renal failure, elevated serum cefepime concentrations increase CNS exposure through a dual mechanism: diminished renal elimination raises the systemic concentration gradient driving passive entry into the CNS, while the concurrent accumulation of endogenous organic acids and uremic toxins competitively inhibits Organic Anion Transporter 3 at the choroid plexus and cerebral endothelium, impairing active efflux of cefepime from the cerebrospinal fluid. This functional blockade of efflux results in drug sequestration within the CNS compartment, with studies demonstrating that although the normal cerebrospinal fluid (CSF)-to-serum concentration ratio is approximately 10%, this ratio may exceed 45% in patients with renal dysfunction^[13]^. The patient’s concurrent sepsis likely amplified this process through cytokine-mediated endothelial injury, further increasing blood–brain barrier permeability and facilitating greater drug accumulation^[14]^. Additional risk factors implicated in CIN are underlying brain abnormalities^[15]^ and old age^[16]^.

Several cases of CIN have been reported, most of which had renal impairment as the most significant causative factor along with higher doses of drug administration. Our patient treated for UTI with cefepime had renal impairment owing to acute kidney injury secondary to septic shock, which resulted in decreased drug clearance. Her previous stroke and old age also contributed to the neurotoxic effects. A similar case was reported in which an elderly patient with chronic renal dysfunction was treated with cefepime for a UTI caused by multidrug-resistant bacteria. The patient developed altered mental status with aphasia and muscle cramps on the second day and had to be treated with hemodialysis^[17]^. In June 2012, the United States Food and Drug Administration sent out a safety announcement reminding clinicians to adjust the doses of cefepime in patients with renal impairment due to possible seizure activity as an adverse event^[18]^.

Some other differential diagnoses were considered, such as hospital-acquired delirium, subclinical seizures, stroke, and shunt malfunction. The presence of a VP shunt introduces substantial diagnostic complexity in patients presenting with altered mental status, as neurosurgical reasoning often defaults to the principle that “it is the shunt until proven otherwise”^[19]^. While this bias is protective against missing life-threatening hydrocephalus, it can also lead to diagnostic anchoring and delayed recognition of medical toxicities. Mechanical shunt failure classically presents with signs of raised intracranial pressure, including headache, vomiting, and lethargy^[20]^, but may also manifest insidiously with non-specific cognitive or behavioral changes that closely resemble metabolic encephalopathy. This overlap was particularly relevant in our patient, who had a history of over-shunting and slit ventricle syndrome, a condition characterized by chronically small, collapsed ventricles due to reduced brain compliance^[21]^, where ventricular enlargement may not occur despite obstruction. In this context, neuroimaging offered limited discriminatory value, and EEG emerged as the definitive diagnostic modality. The patient’s EEG demonstrated generalized slowing with frequent generalized triphasic waves (TWs), a pattern historically associated with hepatic encephalopathy but now recognized as a non-specific marker of moderate-to-severe toxic–metabolic encephalopathy. TWs are believed to arise from functional disruption of thalamocortical relay circuits, wherein toxic or metabolic insults hyperpolarize thalamic neurons, inducing synchronized oscillatory cortical activity^[22]^. Importantly, TWs are consistently reported as the hallmark EEG finding in CIN and often precede or coexist with nonconvulsive status epilepticus (NCSE). Differentiating between these entities was critical, as cefepime is a known precipitant of NCSE in up to 31% of CIN cases^[3]^. Although TWs may morphologically resemble epileptiform discharges, the absence of EEG evolution in frequency or morphology, as defined by the Salzburg criteria, argued against NCSE in this case^[23]^. This distinction allowed clinicians to avoid unnecessary aggressive anticonvulsant therapy and instead focus on the removal of the offending agent. The subsequent resolution of EEG abnormalities and clinical symptoms following cefepime discontinuation retrospectively confirmed the diagnosis of reversible toxic encephalopathy rather than a primary epileptogenic process.

Regarding management, identifying risk factors associated with CIN is imperative, and drugs implicated should be avoided or dosage adjusted according to renal function. The cornerstone of management is prompt discontinuation of cefepime, which represents the single most decisive and effective intervention, with substitution of a non-beta-lactam antibiotic when appropriate. Clinical improvement typically begins within 24–48 hours of drug cessation, with complete resolution of altered mental status occurring within up to 72 hours as cefepime gradually clears from the CNS compartment^[3]^. In cases of non-resolution, continuous EEG monitoring should be performed in conjunction with other options, such as anti-epileptic drugs, such as lorazepam and phenytoin^[24]^. In patients with severe renal impairment, cefepime pharmacokinetics are markedly altered, with the drug’s half-life prolonged from approximately 2 hours to more than 13–20 hours, significantly increasing the risk of sustained neurotoxicity. Although our patient improved with supportive care alone, hemodialysis remains the most effective rescue therapy in cases of severe toxicity, such as coma or refractory NCSE, as a single dialysis session can remove substantial amounts of cefepime and result in rapid neurological recovery^[25]^. The decision to forego dialysis in this case suggests either sufficient residual renal function to permit drug clearance following cessation or early recognition of toxicity before profound CNS accumulation. Our patient’s mental status improved after discontinuation of cefepime, and she was advised regular follow-up for continued monitoring of neurological symptoms.

A primary limitation of this report is the unavailability of therapeutic drug monitoring; specifically, serum or CSF cefepime concentrations were not quantified during the acute phase of encephalopathy. Consequently, the diagnosis of CIN remained presumptive, relying on the exclusion of alternative etiologies and the temporal correlation between drug discontinuation and clinical recovery rather than confirmation via toxicokinetic thresholds.

## Conclusion

While the association between renal impairment and CIN is established, this case highlights the specific diagnostic challenge imposed by concurrent neurosurgical comorbidities. In patients with VP shunts and slit ventricle syndrome, clinical suspicion is frequently diverted toward mechanical failure, which leads to diagnostic anchoring. This report demonstrates that CIN can clinically simulate structural shunt dysfunction and necessitates considering antibiotic toxicity as a primary differential. Furthermore, this case validates the utility of EEG as a decisive tool to prevent unnecessary neurosurgical interventions in patients with multi-hit physiology involving sepsis and renal dysfunction. Consequently, the threshold for early EEG and discontinuation of high-risk beta-lactams must be lowered in complex neurosurgical patients even when mechanical hardware provides a plausible alternative explanation for neurological decline.

## Data Availability

The datasets are available from the corresponding author on reasonable request.

## Research registration unique identifying number (UIN)

This is not an original research project involving human participants in an interventional or an observational study but a case report. This registration was not required.

## AI use declaration

Generative AI was used solely to support the writing process by assisting with grammar and spelling checks, improving clarity during drafting, and refining grammar and flow during revisions, with no role in data analysis or figure creation. Grammarly (web-based, version 14.1249.0) and ChatGPT (OpenAI GPT-5 model family via chat.openai.com under default parameters) were employed exclusively for grammar correction, clarity enhancement, and text revision, with no plug-ins, fine-tuning, or system integrations. No patient text or de-identified images were used as AI inputs, and all inputs were compliant with GDPR/HIPAA standards. All AI-assisted outputs were reviewed, edited, and approved by the supervising authors, Muhammad Furqan and Muhammad Usman Haider, who assume full responsibility for the final content. AI tools were used only for language refinement, with adherence to ethical standards, and transparency was maintained by using simple text-based prompts without creating complex prompts, coding, or shareable artifacts.

## References

[1] Paulvannan V Lakshmikanthcharan S Vivekananthan P. Cephalosporins-A friend or foe? An uncommon cause for encephalopathy: a case report. J Anaesth Crit Care Case Rep 2020;6:17–19.

[2] Taylor J Gunter HM Cohen K. Cefepime-induced neurotoxicity. S Afr J Infect Dis 2025;40:704. 40357178 10.4102/sajid.v40i1.704 PMC12067639

[3] Appa AA Jain R Rakita RM. Characterizing cefepime neurotoxicity: a systematic review. Open Forum Infect Dis 2017;4:ofx170. 29071284 10.1093/ofid/ofx170 PMC5639733

[4] Boschung-Pasquier L Atkinson A Kastner LK. Cefepime neurotoxicity: thresholds and risk factors. A retrospective cohort study. Clin Microbiol Infect 2020;26:333–39. 31284030 10.1016/j.cmi.2019.06.028

[5] Payne LE Gagnon DJ Riker RR. Cefepime-induced neurotoxicity: a systematic review. Crit Care 2017;21:276. 29137682 10.1186/s13054-017-1856-1 PMC5686900

[6] CARE Case Report Guidelines. CARE Checklist. Accessed 26 Aug 2025. https://www.care-statement.org/checklist

[7] Riaz AA Ginimol M Rasha R. Transparency in the reporting of Artificial Intelligence – the TITAN guideline. Prem J Sci 2025;2:1–2.

[8] Bazan JA Martin SI Kaye KM. Newer beta-lactam antibiotics: doripenem, ceftobiprole, ceftaroline, and cefepime. Infect Dis Clin North Am 2009;23:983–96,ix. 19909894 10.1016/j.idc.2009.06.007

[9] Roger C Louart B. Beta-lactams toxicity in the intensive care unit: an underestimated collateral damage? Microorganisms 2021;9:1505. 34361942 10.3390/microorganisms9071505 PMC8306322

[10] Fugate JE Kalimullah EA Hocker SE. Cefepime neurotoxicity in the intensive care unit: a cause of severe, underappreciated encephalopathy. Crit Care 2013;17:R264. 24200036 10.1186/cc13094 PMC4057506

[11] Ilechukwu ST. Acute psychotic reactions and stress response syndromes following intramuscular aqueous procaine penicillin. Br J Psychiatry 1990;156:554–59. 2386866 10.1192/bjp.156.4.554

[12] Guo SB Meng Y Lin L. Artificial intelligence alphafold model for molecular biology and drug discovery: a machine-learning-driven informatics investigation. Mol Cancer 2024;23:223. 39369244 10.1186/s12943-024-02140-6 PMC11452995

[13] Lee SJ. Cefepime-induced neurotoxicity. J Neurocrit Care 2019;12:74–84. 10.1007/s12028-019-00682-y 30756319

[14] Stratton K Davis KW. Case report: cefepime induced neurotoxicity following a change in infusion time. Hosp Pharm 2024;59:411–14. 38919756 10.1177/00185787241237142 PMC11195832

[15] Grill MF Maganti R. Cephalosporin-induced neurotoxicity: clinical manifestations, potential pathogenic mechanisms, and the role of electroencephalographic monitoring. Ann Pharmacother 2008;42:1843–50. 19033476 10.1345/aph.1L307

[16] Mattappalil A Mergenhagen KA. Neurotoxicity with antimicrobials in the elderly: a review. Clin Ther 2014;36:1489–1511.e4. 25450476 10.1016/j.clinthera.2014.09.020

[17] Kim SY Lee IS Park SL. Cefepime neurotoxicity in patients with renal insufficiency. Ann Rehabil Med 2012;36:159–62. 22506251 10.5535/arm.2012.36.1.159 PMC3309312

[18] Research C for DE and. FDA drug safety communication: cefepime and risk of seizure in patients not receiving dosage adjustments for kidney impairment. FDA [Internet]. 2019 Accessed 2025 Aug 26. https://www.fda.gov/drugs/drug-safety-and-availability/fda-drug-safety-communication-cefepime-and-risk-seizure-patients-not-receiving-dosage-adjustments

[19] Albright L Pollack IF Adelson D. eds.. Principles and Practice of Pediatric Neurosurgery [Internet]. Stuttgart: Georg Thieme Verlag; 2008. Accessed 2025 Dec 16. http://www.thieme-connect.de/DOI/DOI?10.1055/b-002-57134

[20] Ransom ER Komotar RJ Mocco J. Shunt failure in idiopathic intracranial hypertension presenting with spontaneous cerebrospinal fluid leak. J Clin Neurosci 2006;13:598–602. 16678427 10.1016/j.jocn.2005.08.008

[21] Panagopoulos D Karydakis P Themistocleous M. Slit ventricle syndrome: historical considerations, diagnosis, pathophysiology, and treatment review. Brain Circ 2021;7:167–77. 34667900 10.4103/bc.bc_29_21 PMC8459697

[22] Liu C Cheng S Ma Y. Triphasic waves in electroencephalogram as a possible early marker of carcinomatous meningitis: a case report. Medicine (Baltimore) 2020;99:e21735. 32872059 10.1097/MD.0000000000021735 PMC7437808

[23] Rachael M Keira M Paul C. In: Journal of Neurology, Neurosurgery & Psychiatry. Non-convulsive status epilepticus or triphasic encephalopathy? 2023;A49.

[24] Grill MF Maganti RK. Neurotoxic effects associated with antibiotic use: management considerations. Br J Clin Pharmacol 2011;72:381–93. 21501212 10.1111/j.1365-2125.2011.03991.x PMC3175508

[25] Chatellier D Jourdain M Mangalaboyi J. Cefepime-induced neurotoxicity: an underestimated complication of antibiotherapy in patients with acute renal failure. Intensive Care Med 2002;28:214–17. 11907668 10.1007/s00134-001-1170-9

